# Sulfidic Habitats in the Gypsum Karst System of Monte Conca (Italy) Host a Chemoautotrophically Supported Invertebrate Community

**DOI:** 10.3390/ijerph19052671

**Published:** 2022-02-25

**Authors:** Giuseppe Nicolosi, Sandro Galdenzi, Maria Anna Messina, Ana Z. Miller, Salvatore Petralia, Serban M. Sarbu, Marco Isaia

**Affiliations:** 1 Department of Life Sciences and Systems Biology, University of Turin, 10124 Torino, Italy; marco.isaia@unito.it; 2 Centro Speleologico Etneo, 95123 Catania, Italy; messina.marianna@gmail.com (M.A.M.); salvatore.petralia@unict.it (S.P.); 3 Scuola di Scienze e Tecnologie—Sezione di Geologia, Università di Camerino, 62032 Camerino, Italy; galdenzi.sandro@tiscali.it; 4 Instituto de Recursos Naturales y Agrobiología de Sevilla, IRNAS-CSIC, 41012 Sevilla, Spain; anamiller@irnas.csic.es; 5 HERCULES Laboratory, University of Évora, 7000-809 Evora, Portugal; 6 Dipartimento di Scienze del Farmaco e della Salute, University of Catania, 95124 Catania, Italy; 7 Emil Racoviţă Institute of Speleology, Str. Frumoasa nr. 31, 010986 Bucureşti, Romania; serban.sarbu@yahoo.com

**Keywords:** gypsum cave, stable isotope ecology, food web analysis, cave-dwelling fauna, sulfide, ecosystem conservation

## Abstract

The great diversity of the invertebrate community thriving in the deepest sections of the gypsum karst system of the Monte Conca sinkhole (Sicily, Italy) suggests the existence of a complex food web associated with a sulfidic pool and chemoautotrophic microbial activity. To shed light on the peculiarity of this biological assemblage, we investigated the species composition of the invertebrate community and surveyed trophic interactions by stable isotope analysis. The faunal investigation conducted by visual censuses and hand sampling methods led to the discovery of a structured biological assemblage composed of both subterranean specialized and non-specialized species, encompassing all trophic levels. The community was remarkably diverse in the sulfidic habitat and differed from other non-sulfidic habitats within the cave in terms of stable isotope ratios. This pattern suggests the presence of a significant chemoautotrophic support by the microbial communities to the local food web, especially during the dry season when the organic input from the surface is minimal. However, when large volumes of water enter the cave due to local agricultural activities (i.e., irrigation) or extreme precipitation events, the sulfidic habitat of the cave is flooded, inhibiting the local autotrophic production and threatening the conservation of the entire ecosystem.

## 1. Introduction

In terms of energy inputs, subterranean ecosystems are generally regarded as oligotrophic environments, especially in the inner and most isolated parts [1]. Nutrient supplies mainly depend on the flux of energy from the surface, which enters the subterranean habitats in a variety of ways: carried by gravity from the cave entrances and ceiling cracks, by water, wind, or by animals such as bats, birds or rodents [1,2,3,4,5,6].

Several studies have underlined how some subterranean ecosystems do not depend entirely on organic matter coming from the surface [7,8,9,10,11,12,13] but instead, on chemoautotrophic in situ production of organic matter by microorganisms processing different inorganic elements to obtain energy and nutrients. These organisms represent de facto the only possible primary producers inhabiting the subterranean ecosystem. In some cases, such non-photoautotrophic subterranean biological assemblages support unusually rich local biological communities, even when they are completely isolated from the surface [14,15,16,17]. Moville Cave in Romania represents one of the most interesting and well known examples of a chemoautotrophic hypogean system, characterized by hydrogen sulfide-rich groundwater [18]. As demonstrated by carbon and nitrogen stable isotope analyses [7], the cave is a closed system, fully supported by chemosynthesis. Here Vlasceanu et al. [19] reported the presence of *Thiobacillus thioparus*, a bacterium capable of oxidizing hydrogen sulfide to sulfuric acid. The organic material produced in situ allows the development of a rich and diversified community composed of different species, 37 of which are endemic of this subterranean groundwater ecosystem [13,18,20,21]. In other cases, chemoautotrophic bacterial activity represents an additional—but not exclusive—energy source in the cave. This is the case of semi-closed hypogean systems such as the Cesspool Cave in USA [16,22].

In this paper, we aimed to describe the biological community dwelling in the Monte Conca sinkhole (W-Sicily, Italy) and to demonstrate the role of the resident chemoautotrophic organisms in the cave food web, providing evidence for their independence from external inputs. For these purposes, we identified the most abundant and detectable species dwelling in the cave, we sorted them in two groups according to their occurrence in sulfidic and non-sulfidic habitats, and we compared the relative abundance of C and N stable isotopes in the two groups by means of Stable Isotope Ratio Analysis (SIRA).

## 2. Materials and Methods

### 2.1. Site Description

**Figure 1:**
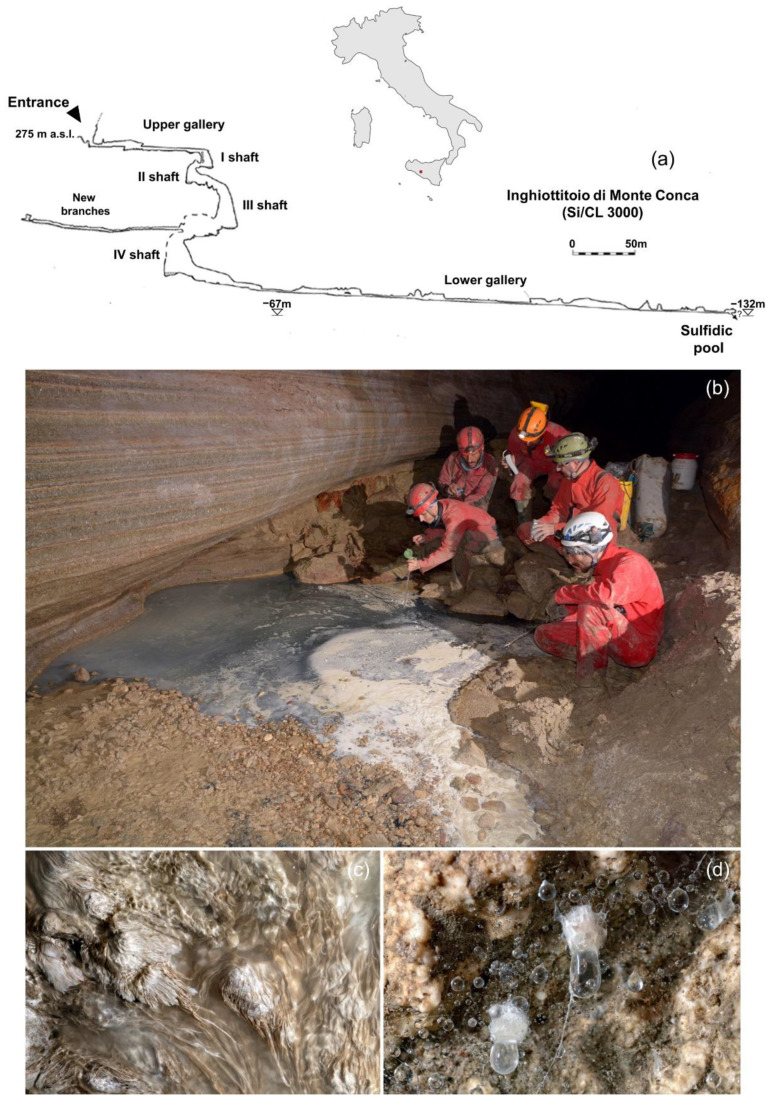
(**a**) Map of the study area (topography of the Monte Conca sinkhole (Adapted with permission from Madonia and Vattano [23]. 2008 Vattano). (**b**) Sulfidic habitat at the end of the lower gallery containing sulfur suspensions on water surface. (**c**) Filamentous microbial mats floating on the sulfide pool surface. (**d**) Acid droplets (snottites) hanging from the ceiling of the cave. Photos: F. Fiorenza.

The Monte Conca sinkhole (cadastral number 3000SI-CL, 37°29′19.7″ N 13°42′46.5″ E) is an active cave developed in Messinian evaporites occurring in central western Sicily (Italy), within the Strict Nature Reserve of “Monte Conca”. It is also a Geosite of regional interest, only accessible based on authorized permission. Currently, Monte Conca sinkhole is considered the longest and deepest gypsum karst system in Sicily, reaching a depth of 130 m and a total passage development of more than 2.5 km [23]. The entrance gallery is about 100 m long, followed by four shafts of 11, 12, 35 and 26 m deep, respectively (Figure 1a). At the bottom of the fourth shaft, a 450 m long gallery leads to the terminal part of the cave, where a sulfidic pool is located, fed by a stream seeping from bedrock (Figure 1b). Physical and chemical analyses conducted by previous researchers [24] have provided robust evidence for the presence of bacterial activity, as witnessed by filamentous microbial mats floating on the water surface of the sulfidic waters (Figure 1c) and by organic stalactites (snottites) on the cave walls and ceilings (Figure 1d), rich in sulfuric acid (H_2_SO_4_). Davis et al. [25] documented the presence of the microbial community dominated by sulfur-oxidizing bacteria.

The cave floor is predominantly bedrock, frequently covered by the stream. Sediments of different origin such as clay, mud and gravel and chemical deposits (iron and gypsum deposits), are visible in the lower parts of the gallery.

During the wet season—generally from January until May—large volumes of water enter the cave from a tributary of the Gallodoro stream, flooding it entirely [24] and mostly precluding access to the cave. Such floods result in massive inputs of allochthonous surface materials into the cave as testified by remarkable accumulations of mud and organic matter in the lower galleries that may even hamper speleological progression. Surface organic debris also percolate through the fractured ceiling, providing an additional source of external organic carbon. Water derived from surface-runoff during the wet season also facilitates the entry of anthropogenic microbes, including potential contaminants, such as *Escherichia* and *Lysinibacillus* bacteria which are likely derived from outside, particularly from the agricultural fields located above the cave [25].

In July and August, i.e., the dry season, the stream dries out and no water flows inside the cave, leaving only small and isolated water pools. According to recent microbiological studies [25], such conditions primary favor sulfur-oxidizing bacteria such as *Sulfurovum*, *Sulfurimonas*, *Thiovirga* and *Arcobacter*. However, sudden extreme meteorological events during summer may fill up the cave altering these peculiar conditions, besides shutting the way out to speleologists and making investigations in the cave particularly hazardous.

### 2.2. Habitat Characterization

**Figure 2:**
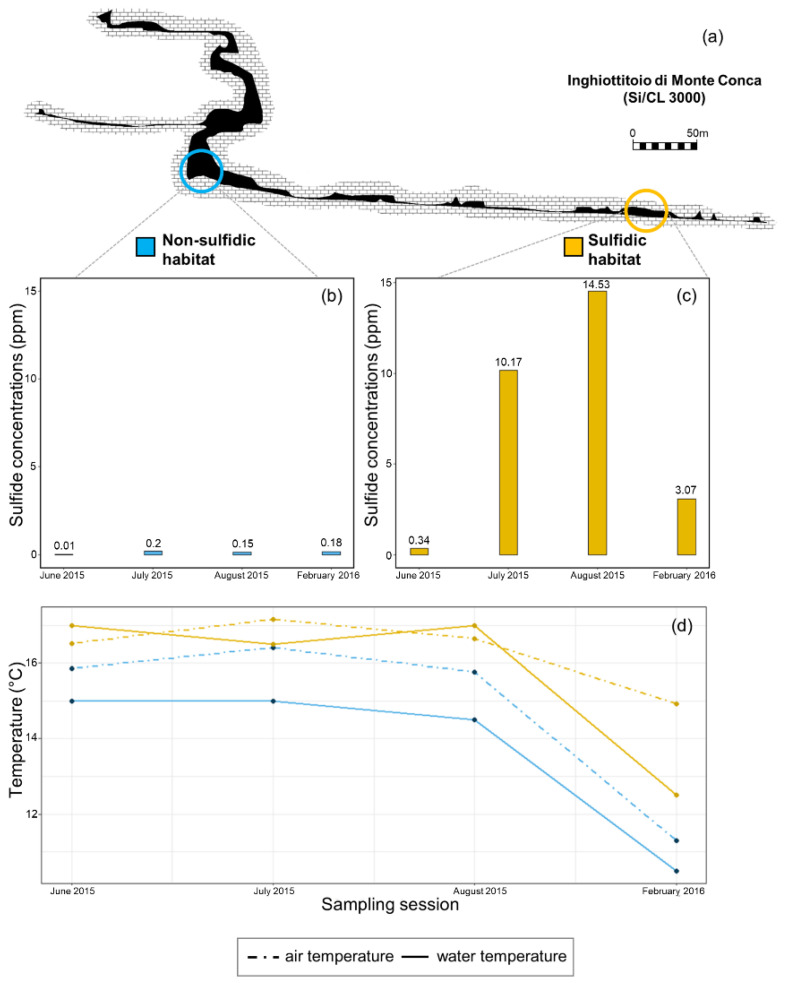
(**a**) Map of the study area with the position (circles) of the sampling sites: blue (non-sulfidic habitat) and orange (sulfidic habitat). Topography of the Monte Conca sinkhole (Adapted with permission from Madonia and Vattano [23]. 2008 Vattano). (**b**) Sulfide concentrations (ppm) in the non-sulfidic and sulfidic (**c**) habitats monitored across four sampling sessions. (**d**) Air and water temperature in Monte Conca sinkhole recorded during the sampling session (June, July, August 2015, and February 2016) at non-sulfidic and sulfidic habitats.

Habitats along the cave were characterized by means of chemical (pH and sulfide concentration) and physical (water and air temperature) parameters. The measurements were performed in a non-sulfidic control pool located under the fourth shaft (non-sulfidic habitat, blue circle in Figure 2a) and in a pool in the terminal gallery (sulfidic habitat, yellow circle in Figure 2a).

The sulfide concentration in the water was measured by Cline’s methylene blue method [26]. For this purpose, 10 mL of water for each sample were stabilized with 1.5 mL of Zn acetate. The solution was added with a volume of 10 mL of N,N, dimethyl–p-phenylendiammonium solution and 1 mL of iron(III) chloride solution. Once in the laboratory, after 30 min of stirring at room temperature, the absorbance value at 666 nm was measured for each sample by a Molecular Devices SpectraMax^®^ spectrophotometer. The amount of sulfide was then calculated using calibration lines in the range from 0.1 to 20 ppm. Sulfide concentration was measured at the two sampling sites (control and sulfidic pools) in four sampling sessions (June, July, August 2015, and February 2016).

Air and water temperature were measured by using a HOBOware sensor (sensitivity, 0.01 °C) and a CM-35 Crison multimeter probe respectively. Measurements were taken during each of the four sampling sessions in both the sulfidic and control pools.

### 2.3. Biological Survey

To obtain an accurate knowledge of the biological community dwelling in the cave, we conducted four surveys, two in the dry and two in the wet season (Table 1). The high level of risk associated with the access and the permanence in the cave precluded us from using standardized sampling methodologies (i.e., pitfall trapping) that would allow us to examine abundance trends in invertebrates. Accordingly, both the aquatic and terrestrial community were investigated in terms of presence/absence data.

Species were monitored through visual census, although identification often required the collection of specimens.

Terrestrial invertebrates were collected manually using tweezers, searching walls, floor, ceiling, and turning over rocks and debris. Aquatic macroinvertebrates were collected using a needle-less syringe (60 mL) in the pools and a fine mesh net (60 micron) along the stream. Additionally, we used bottle traps to collect amphipods.

All specimens were sorted under a stereomicroscope and identified to the lowest possible taxonomic level. Material was preserved in 70% ethanol. For certain groups requiring DNA analysis (i.e., Clitellata), we preserved specimens in 95% ethanol.

Identifications were supported by specialists (Gastropoda, Clitellata, Copepoda, Amphipoda) (see Acknowledgements). A number of specimens (Ostracoda) could not be identified to the species level. Nomenclature for all groups follows the Global Biodiversity Information Facility database [27].

### 2.4. Stable Isotope Analysis

We used Stable Isotope Ratio Analysis (SIRA) of carbon and nitrogen to determine the level of independence from the surface of the food-web as suggested by Sarbu et al. [7] (see also De Niro and Epstein [28], Vlasceanu et al. [29], Engel et al. [30], Paoletti et al. [31] and Michener and Lajtha [32]. SIRA represents an effective tool to determine food sources in a given ecosystem because organisms fractionate isotopes of carbon (^13^C/^12^C) and nitrogen (^15^N/^13^N) in predictable ways. For this purpose, we only focused on the organisms collected in the dry season i.e., when maximal isolation from the surface is achieved. Samples were assigned to the “sulfidic habitat” category when collected within or in close proximity to the sulfidic pools, and to the “non-sulfidic habitat” when collected within, or in close proximity to, the control pool (Figure 2a).

Analyses were performed on the most detectable species. Samples were placed in falcon tubes and, once in laboratory, washed with deionized water and dried. Samples of *Nepa cinerea* were also brushed to remove the bacterial coated layer formed within the sulfidic pool. Samples of *Tubifex blanchardi* and *Proasellus montalentii* were composed of several individuals to guarantee sample size for mass spectrometry. Large macroinvertebrates were analyzed individually.

Moreover, we collected a sample of the white biofilm in the sulfidic pool as this could represent the food source for a chemoautotrophically-based ecosystem. For comparison, a sample of organic matter was also collected in the non-sulfidic habitat (i.e., decaying plant and other organic remains of surface origin).

Isotopic composition was determined using an isotope mass spectrometer. Stable isotope data are presented in the delta (δ) notation as the relative difference between the ratios of the sample and the standards: δ^13^C = [(^13^C/^12^C) − 1] × 1000 and δ^15^N = [(^15^N/^14^N) − 1] × 1000, where δ^13^C or δ^15^N are reported in part per thousand (‰). Atmospheric N_2_ is the standard for nitrogen, while Vienna PeeDee belemnite (VPDB) is the standard for carbon.

The Stable Isotope Ratio Analysis was performed at the University of New Mexico, Department of Earth and Planetary Sciences, Albuquerque, NW, USA.

To test for the actual separation between the sulfidic and non-sulfidic habitats, δ^13^C values were statistically compared with the Student’s *t*-test. For this purpose, only the species shared by the two habitats were analyzed. Normality was at first tested using the ‘shapiro.test’ function from ‘stats’ package, ver. 4.2.0 [33]. The F-test was used to check for homogeneity in variances by using the ‘var.test’ function from ‘stats’ package. The main test was performed using the ‘t.test’ function from ‘stats’ package.

## 3. Results

### 3.1. Habitat Characterization

Sulfide concentrations were negligible (0.03 ppm and 0.18 ppm) in the control plot (Figure 2b). Values of H_2_S in the sulfidic habitat (Figure 2c) were generally higher, ranging from 0.34 ppm (June) to 14.53 ppm (August).

Air and water temperatures in the control site were relatively stable in June, July and August (around 16 °C and 15 °C, respectively). Temperature dropped in February, reaching 11.5 °C and 10.5 °C, respectively. (Figure 2d).

Air and water temperature in the sulfidic habitats followed the same trend across the year, but values were generally higher than those measured in the non-sulfidic habitat, with values at least 0.6 °C higher (June). The highest deviation was +3.6 °C, recorded in February (Figure 2d). Water temperature differed between the sulfidic and non-sulfidic habitats, with +1.5 °C in July 2015 and +2.5 °C in August (Figure 2d).

### 3.2. Biological Survey

The biological survey determined the presence of 54 species (Table 2, see also Supplementary Material (Table S1) for the complete species list), including 27 species collected at the cave entrance, 39 in the non-sulfidic habitat and 48 in the sulfidic one. However, just a few of them showed subterranean adaptations, thus demonstrating a general epigean origin of the invertebrate assemblage colonizing the cave. Some of the species, such us the water scorpion *Nepa cinerea* or the beetle *Paranchus albipes* were found in the dry and the wet season, hinting at their possible presence in the cave throughout the year. The general epigean origin of the assemblage is related to passive water transportation during the wet season, contributing to an overall and seasonal increase of the species diversity at the inner parts of the cave. Predators were highly diversified in the assemblage (24 species), including 18 species at the cave entrance, 16 in the non-sulfidic habitat and 20 in the sulfidic one. Detritivores were represented by 20 species, (16 at the cave entrance, 20 in the non-sulfidic habitat and 20 in the sulfidic one).

During the dry season, the lack of surface water flowing in the cave reduces the intake of epigean species, drastically reducing the overall number of species in the cave. Predators drop dramatically in both sulfidic (9) and non-sulfidic habitats (7), as well as the number of detritivores (14 and 7 species respectively), that become the most diverse group in the sulfidic habitat (Table 2).

**Figure 3:**
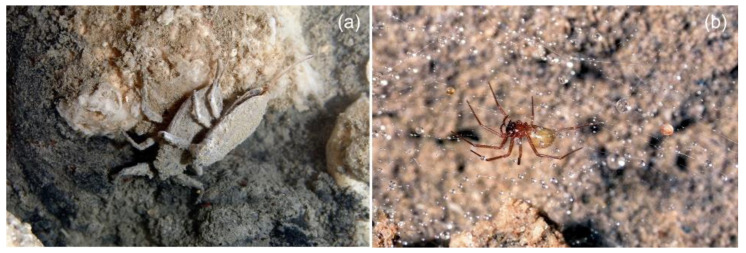
(**a**) The water scorpion *Nepa cinerea* dwelling in the sulfidic waters. (**b**) The spider *Lessertia barbara* on its webs with droplets of sulfuric acid (pH ~1). Photos: F. Fiorenza.

The aquatic invertebrate community was remarkably diverse, especially in the sulfidic habitat where we detected the presence of numerous species in all trophic levels, including detritivores such as *Pseudamnicola (Pseudamnicola) moussonii* (Gastropoda, Hydrobiidae), *Proasellus montalentii* (Malacostraca, Asellidae) and *Tubifex blanchardi* (Clitellata, Naididae). Among predators, we detected *Haemopis sanguisuga* (Clitellata, Haemopidae) and *Nepa cinerea* (Insecta, Nepidae) (Figure 3a). The latter was also present in the control habitat, but in lower numbers.

Among terrestrial species, spiders were usually found under rocks, dead wood or in crevices in the walls. Some were only present in the twilight zone of the cave (e.g., *Metellina merianae*, *Holocnemus pluchei*), while others, such as the troglophile *Kryptonesticus eremita*, were also found dwelling on organic debris and on cave walls in the terminal section of the cave, in both sulfidic and non-sulfidic habitats.

The spider *Lessertia barbara* was spotted dwelling on some heaps of organic material in proximity of the sulfidic pools (Figure 3b). Here, we noticed the presence of the spider egg sacs hanging from the spider webs among droplets of sulfuric acid (pH ~1) sticking on the web threads (Figure 3b).

### 3.3. Stable Isotope Analysis

Stable isotope analysis was performed on the most detectable biological species. For the sulfidic habitat we analyzed *n* = 3 *Agabus* sp. (Insecta, Coleoptera), *n* = 1 larva and 1 adult of *Meladema coriacea* (Insecta, Coleoptera), *n* = 4 *Kryptonesticus eremita* (Arachnida, Araneae), *n* = 3 *Nepa cinerea* (Insecta, Hemiptera), *n* = 3 *Paranchus albipes* (Insecta, Coleoptera), *n* = 1 sample of *Proasellus montalentii* (Malacostraca, Asellidae), *n* = 1 sample of *Tubifex blanchardi* (Clitellata, Haplotaxida). For the non-sulfidic habitat, we analyzed *n* = 1 larva Dytiscidae (Insecta, Coleoptera), *n* = 1 Julidae (Diplopoda), *n* = 5 *Kryptonesticus eremita*, *n* = 1 *Meladema coriacea*, *n* = 2 *Nepa cinerea*, *n* = 1 *Oxychilus lagrecai* (Gastropoda, Stylommatophora).

**Figure 4:**
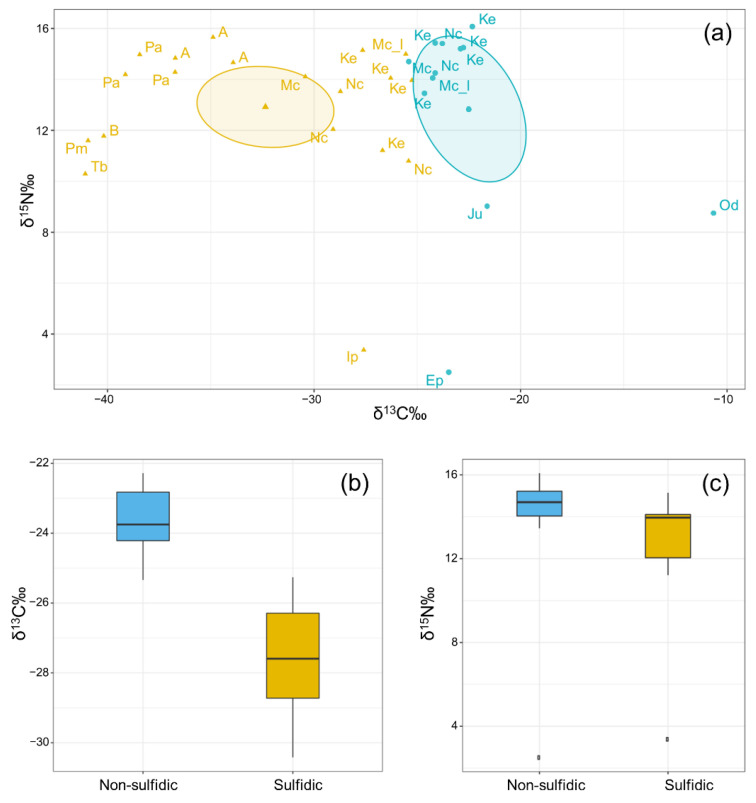
(**a**) Scatter plot of δ^15^N against δ^13^C in sulfidic (yellow) and non-sulfidic (blue) habitat. The samples differ in their δ^13^C, indicating differential use of carbon sources. Abbreviations: A = *Agabus* sp., B = biofilm, Oc = organic matter collected in the control pool (non-sulfidic habitat), Ju = Julidae, Ke = *Kryptonesticus eremita*, Nc = *Nepa cinerea*, Mc = *Meladema coriacea*, Mc_l = *Meladema coriacea* (larvae), Od = *Oxychilus lagrecai*, Pa = *Paranchus albipes*, Pm = *Proasellus montalentii*, Tb = *Tubifex blanchardi*. (**b**) Boxplot of the δ^13^C‰ values referred to sulfidic vs. non-sulfidic habitat. (**c**) Boxplot of the δ^15^N‰ values referred to sulfidic vs. non-sulfidic habitat.

The results of isotope analysis revealed the presence of an autochthonous food source. In particular, the aquatic biofilm collected in the sulfidic habitat (presumably sulfur-oxidizing microorganisms) was isotopically light, showing a δ^13^C value of −40.18‰ and a δ^15^N value of 11.77‰, indicating a chemoautotrophic food source. This differed significantly from the organic matter collected in the control habitat, where values of δ^13^C were −23.46‰ (Figure 4a), indicating differential use of carbon sources. The C:N ratio for the white biofilm was 4.8, in accordance with white filament bundles observed in Lower Kane Cave (C:N ratios ~5), suggesting a high-quality food source [16]. Contrarily, the C:N value for the organic matter in the control pond was higher, reaching 8.2–13 attesting the lower quality of the food source.

Samples collected in the sulfidic habitat were isotopically lighter in carbon (δ^13^C = −41.08 to −25.26‰) but rather similar in nitrogen (δ^15^N = 10.29 to 15.66‰) than the one collected in the non-sulfidic habitat (Figure 4a). The latter were isotopically heavier in carbon (δ^13^C = −10.64 to −25.34‰) but similar in nitrogen (δ^15^N = 8.75 to 16.08‰). For instance, the δ^13^C value for *Tubifex blanchardi* and *Proasellus montalentii* were −41.078‰ and −40.94‰, respectively. Both *T. blanchardi* and *P. montalentii* likely feed on microbial mats as their δ^13^C values differ considerably from the other invertebrates collected in the sulfidic habitat (Figure 4a). This interpretation is consistent with Rodriguez et al. [34] having documented for *T. blanchardi* the use of bacteria as a food resource.

The beetles *Agabus* sp. and *Paranchus albipes* showed isotopically lighter values of δ^13^C (−35.17 and −30.10‰, respectively). As both are predators, they probably feed on organisms isotopically lighter such as the isopod *Proasellus montalentii*.

Species present in both the sulfidic and the control habitat, such as the bug *Nepa cinerea*, the spider *Kryptonesticus eremita* and the beetle *Meladema coriacea*, allowed a direct comparison of their isotopic values and C:N ratios. For these specimens, the δ^13^C values were also isotopically heavier in the control habitat (respectively −23.93, −23.29, −25.33‰) compared to the sulfidic one (−27.74, 26.47, −30.42‰). The C:N ratio values were similar in both habitats. Predators in the non-sulfidic habitat *Nepa cinerea, Kryptonesticus eremita* and *Meladema coriacea* had a value of 4.2, 4.1 and 3.9, respectively, whereas in the sulfidic habitat, their values were 4.2, 4.3 and 4.3, respectively.

Differences among the two habitats in terms of δ^13^C values were statistically significant (t = −5.75, df = 16, *p*-value =< 0.001) confirming a clear separation of the two habitats, at least during the dry period (Figure 4b). Values of δ^15^N were comparable in the two habitats (Figure 4c).

## 4. Discussion

The Monte Conca sinkhole is a gypsum karst system containing a sulfidic pool with documented microbial chemoautotrophic activity [24].

The number of subterranean invertebrate species in Monte Conca sinkhole is rather low if compared with other caves with similar chemical-physical characteristics, such as the Frasassi caves in Italy and Movile Cave in Romania [13,35].

The intermittent action of the stream favors the accidental introduction of surface-dwelling invertebrates in the cave during the wet season. Consequently, surface organisms that are transported in the lower galleries of the cave can temporarily colonize the terminal section of the cave. In contrast, during the dry season their numbers decrease drastically, nearly disappearing.

On the one hand, the presence of a dry season prevents the entrance of accidental species, yet on the other, it seems to favor the resident cave fauna, especially those organisms dwelling in the sulfidic habitat. Our results suggest that the existence of a non-photosynthetic food source sustains a high biodiversity in the cave, especially in the sulfidic ponds. The great diversity of predators and omnivores suggests the presence of a more complex trophic web compared to non-sulfidic habitats within the cave, where the nutrient supplies likely have an allochthonous (epigean) origin. Remarkably, the number of species of detritivores in the sulfidic habitat was twice as high as in the control habitat. It seems likely that such diversity is favored by the high quantity and quality of the white autotrophic microbial biofilm representing the base of the food web in the sulfidic habitat.

During the dry season, dense clusters of *T. blanchardi* dwell in the H_2_S-rich water of the sulfidic pool. Here, they probably proliferate facilitated by their ability to survive for long periods in anoxic, sulfidic or heavily polluted areas (e.g., Volpers and Neumann [36] and Martins et al. [37]) as well as by the great availability of bacteria as food resource [34]. The great availability of prey (i.e., *Proasellus montalentii* and *T. blanchardi*) in the sulfidic pools also parallels the presence of a diversified assemblage of predators. Among others, the water-scorpion *Nepa cinerea* can tolerate moderate levels of pollution [38] and so to thrive in sulfidic waters, as previously documented by several authors [39,40,41,42].

The spider *Lessertia barbara* does not appear to be at disadvantage by the extreme conditions characterizing the sulfidic habitat. The species was previously uniquely known in Italy from another gypsum cave in Sicily (“Grotta dei Panni”, Santa Ninfa, Trapani), [43]. Remarkably, the species has been described on material collected in a cave in Algeria (“Grotte du lac souterrain” near the Hammam Maskhoutine springs [44]), in several caves in southern Spain [45], and one cave in Morocco [46]. Interestingly, all of these caves are characterized by the presence of sulfidic waters.

The occurrence of nesticid and linyphiid spiders (*Kryptonesticus* spp. and *Phanetta subterranea* Emerton 1875) living close to the sulfidic pools and acid droplet on the spider webs were also observed in Movile, Frasassi and Parker caves [16,47].

According to our results, it seems likely that the proliferation and greater richness of the resident fauna in the terminal part of the cave is favored by the high sulfide concentration characterizing cave waters during the dry season. As suggested by Davis et al. [25], in Monte Conca sinkhole the higher concentration of sulfide favors the prevalence of primary sulfur oxidizers in the microbial community, such as *Sulfurovum*, *Sulfurimonas*, *Thiovirga*, and *Arcobacter*, causing the increase in the production of organic carbon chemosynthetically.

The literature describes elaborate food webs of macroinvertebrates based on chemoautotrophic production in several subterranean ecosystems [7,12,13,34,48] and deep-sea hydrothermal vents and seeps [49,50].

In Monte Conca sinkhole, invertebrates can graze on the in situ produced biomass. In fact, the carbon isotope composition of fauna samples showed markedly lighter values from the sulfidic habitat. This was especially true for organisms feeding directly on bacteria and sediments, such as *Proasellus montalentii* and *Tubifex blanchardi*. The same trend was observed in other sulfidic caves where grazers showed δ^13^C values consistent with consumption of the microbial mats (e.g., Sarbu et al. [35] and Galdenzi et al. [51]).

The isotopic values for invertebrates that live in the sulfidic habitat differed significantly from those collected in the non-sulfidic one. Similarly, Roach et al. [52] revealed that most of the carbon and nitrogen obtained by fish from the sulfidic Cueva del Azufre stream derived from in situ chemoautotrophic production, whereas detritus and green algae were the dominant food sources of surface-dwelling fish from non-sulfidic stream habitat.

However, besides the driest period, the flooding during the wet season prevents the establishment of trophic chains exclusively based on chemoautotrophic production. The wet season causes large volumes of water to runoff from the surface and enter the Monte Conca sinkhole. This input facilitates the contact between the sulfidic and non-sulfidic habitats and a strong dilution of the hydrogen sulfide content of the water. The sulfide concentration measured was in fact considerably reduced in the wet season, also in the sulfidic habitat.

Davis et al. [25] detected a major abundance of microorganisms derived from anthropogenic inputs during the wet season, rather than sulfur oxidizing organisms. They included the bacteria *Escherichia* and *Lysinibacillus*, likely derived from the agricultural regions around the Monte Conca sinkhole. As reported by several authors, changes in surface land use, such as agriculture activities or sewerage system in urban areas, might alter the amount and composition of water entering the cave, promoting changes in speleothem growth and in cave microbial diversity [53,54,55]). González-Pimentel et al. [56] revealed a significant input of anthropized agricultural material from the overlying layers into siliceous speleothems from a lava tube in La Palma Island (Spain), inducing changes on the subsurface microbial diversity. In the Monte Conca sinkhole, although included in the Strict Nature Reserve “Monte Conca”, the presence of agricultural activities poses a threat to this ecosystem, favoring the input of contaminants of anthropogenic origin and altering the natural hydrological regime of the sinkhole. Recent observations have highlighted a constant input of water even during the summer period, probably derived from the irrigation system upstream of the cave entrance. Besides, climatic changes i.e., extremization of meteorological events such as sudden violent summer rains and cloudbursts, are likely to have a similar impact on the natural hydrological regime, inhibiting chemoautotrophic productivity due to water dilution.

## 5. Conclusions

Monte Conca sinkhole is a karst system developed in gypsum. It represents one of the few spots in the world that includes a sulfidic pool located within the cave, where the subterranean invertebrate community relies seasonally on autochthonous food produced chemoautotrophically by sulfur-oxidizing bacteria. Accordingly, the Monte Conca sinkhole could be considered an intermediate semi-closed ecosystem, unlike fully closed systems such as the Movile Cave in Romania, and open ones, as found in most sulfidic caves across the world [16,22].

The land use overlying the cave, in particular agricultural activities and extremization of meteorological events pose major threats to this ecosystem, as changes in the natural hydrological regime might increase the amount of water flowing into the cave, favoring the input of anthropogenic pollutants and altering the structure of its unique chemotrophic based food chain. Consequently, the amount of organic matter produced by the sulfur oxidizers, crucial for the structuring of the local biological assemblage, could fade over time. Such alterations could lead to severe disturbance of this exceptional ecosystem, thus threatening its existence. Due to its considerable scientific importance, Monte Conca sinkhole needs immediate and unquestionable protection.

## Figures and Tables

**Table 1:** Calendar of the sampling sessions and meteorological data provided by SIAS (*Servizio Informativo Agrometeorologico Siciliano*, Italy).

Date	Season	Rainfall (mm) of the Previous Month	Number of Rainy Days in the Previous Month	Daily Mean Outside Temperature
20 June 2015	wet	44.2	8	20.28
11 July 2015	dry	0	0	26.16
29 August 2015	dry	18	5	25.5
6 February 2016	wet	117	21	7.55

**Table 2:** Number of invertebrate species sorted in trophic groups (predators, detritivores, and others i.e., omnivores, herbivores or phytophagous) collected in the three sectors of the Monte Conca sinkhole during the wet (W) and the dry (D) season.

Cave Sector	Predators		Detritivores		Others		Total	
	W	D	W	D	W	D	W	D
Entrance	18	9	3	3	6	4	27	16
Non-sulfidic	16	7	16	7	7	5	39	19
Sulfidic	20	9	20	14	8	6	48	29
Total	24	20	20	18	9	8	54	43

## Supplementary Materials

The following supporting information can be downloaded at: https://www.mdpi.com/article/10.3390/ijerph19052671/s1, Table S1: List of taxa collected within the Monte Conca sinkhole.
